# Tomato cell cultures as innovative plant biostimulants: evaluation of its effect on pepper seeds germinated under salinity conditions

**DOI:** 10.3389/fpls.2025.1726488

**Published:** 2025-12-02

**Authors:** José Manuel Martí-Guillén, Sara Esperanza Martínez-Lorente, Begoña Miras-Moreno, María Ángeles Pedreño, Lorena Almagro, Ana Belén Sabater-Jara

**Affiliations:** Department of Plant Biology, Faculty of Biology, University of Murcia, Espinardo, Murcia, Spain

**Keywords:** bioactive compounds, climate change, higher plant-derived biostimulants, tomato biostimulant, tomato cell cultures, pepper seeds, salt stress, seed priming

## Abstract

The edaphoclimatic pressure derived from climate change is challenging modern agriculture by decreasing the yield of crops necessary to feed the increasingly growing world population. In response to these constraints, plant biostimulants have emerged as a promising and environmentally sustainable approach to enhance crop yield and performance under these adverse conditions. Among the different types of biostimulants, higher plant-derived biostimulants (hPDBs) stand out due to their favorable attributes, including low production costs, safety and being environmentally friendly. Furthermore, their rich composition in diverse bioactive compounds positions them as valuable tools for improving plant growth and stress tolerance within sustainable agricultural systems. In this work, tomato cell cultures elicited with 25 mM Me-β-cyclodextrins and 100 µM methyljasmonate were used to produce a novel type of hPDB through biotechnological tools: tomato biostimulant (TB). Comprehensive untargeted metabolomic analysis revealed a distinctive metabolic profile of TB compared to unelicited cell cultures. Among the discriminant metabolites that explain the differences between the chemical profiles, several were fatty acids, as well as phenolic compounds, including flavonoids and other specialized plant metabolites. TB was applied as seed priming in pepper seeds, with the goal of enhancing the germination process under salt stress and obtaining seedlings with improved vigor and physiological quality and less oxidative damage. The results showed an improvement in germination parameters, such as MGT (-10%), GI (+25%) and VI (+47%); and in oxidative stress markers, by decreasing H_2_O_2_ content by -24%, and promoting POX activity by +35%, compared to non-primed seeds subjected to salt stress. Thus, TB constitutes a novel biostimulant with potential to alleviate salt stress in pepper seeds by promoting antioxidant defenses and reducing oxidative damage.

## 1 Introduction

Present-day agriculture is facing unprecedented challenges due to the combined pressure of rapid global population growth and the rise of climate change, limiting crop productivity worldwide (Mittler et al., 2025; Zhang et al., 2025). In particular, between 20% and 33% of irrigated arable land worldwide is affected by salinity, and this percentage is expected to reach 50% by 2050 (Majeed and Muhammad, 2019). Spain is one of the countries most effected by salinity, with 3% of the 3.5 million hectares of irrigated land severely affected by salinity, significantly reducing its agricultural potential (Acosta et al., 2011). High soil salinity generates severe osmotic stress (limiting seed water uptake and cell turgor) and ion toxicity, which disrupts cellular metabolism, generating an increase in reactive oxygen species (ROS), including O_2_^−^ and H_2_O_2_, and leading to oxidative damage (such as lipid peroxidation). These effects typically delay and inhibit germination, decreasing the germination rate and increasing the average germination time under saline conditions, making seedling establishment a critical phase during crop cycle (Ibrahim, 2016).

This is of particular concern for pepper (*Capsicum annuum* L.), a species of great horticultural interest that is salt-susceptible, experiencing severe reductions in yield when exposed to these conditions. In 2023, Spain produced more than 1 million tons of pepper, making it the fifth largest producer of this vegetable in the world (FAO, 2023). Pepper production in Spain is concentrated in the Mediterranean region, more specifically in Murcia-Almería region (Ministerio de Agricultura, Pesca y Alimentación de España, 2023), one of the most susceptible to deterioration due to soil salinization (Acosta et al., 2011).

Seed germination and seedling growth represent crucial stages in the crop cycle, during which plants are highly sensitive to abiotic stress factors. A promising approach to address this challenge is the use of plant biostimulants, natural products that improve nutrient use efficiency, promote abiotic stress tolerance, and enhance crop quality traits. In particular, higher plant-derived biostimulants (hPDBs) have received increasing attention as environmentally friendly tools to support sustainable agriculture, given the necessity to reduce the use of agrochemicals (European Commission, 2019; Martínez-Lorente et al., 2024). These products include protein hydrolysates and plant extract formulations, which modulate plant physiological and metabolic pathways from germination to fruit development (Martínez-Lorente et al., 2024). In this context, seed priming with hPDB stands out as a cost-effective and efficient practice to improve seed performance under adverse conditions. This seed preconditioning allows partial activation of metabolic and defense pathways before germination, resulting in greater vigor, faster emergence, and greater tolerance to stressful conditions (Chen and Arora, 2013).

However, most hPDBs, made from plant raw materials, present serious limitations in their production and commercialization, particularly due to their highly heterogeneous composition. This is due to the soil and climate variability to which plant raw materials are exposed during their life cycle, leading to changes in product effectiveness (Albaladejo-Marico et al., 2025). To overcome these limitations, plant cell culture technologies are emerging as a promising and sustainable alternative. A key advantage of this biotechnological approach lies in its ability to provide a controlled, reproducible, and homogeneous production of bioactive molecules, overcoming dependence on seasonal fluctuations, geographic location, or environmental variability inherent to field-grown plant material. Furthermore, the productivity and diversity of metabolites obtained from plant cell cultures can be significantly increased by elicitation strategies, which stimulate the biosynthesis and accumulation of specific compounds in a wide variety of cell cultures (e.g., *Vitis vinifera*, *Taxus baccata*, *Daucus carota*, *Capsicum annuum*) (Sabater-Jara et al., 2010, 2014b, 2014a; Belchí-Navarro et al., 2013). Taken together, these traits position plant cell culture technologies as a highly attractive alternative for the development of next-generation biostimulants that promote sustainable agriculture. In fact, recent studies by our research group support this concept: hPDBs derived from yellow carrot (Martí-Guillén et al., 2025a) and grapevine (Martí-Guillén et al., 2025b) cell cultures have been observed to promote seed germination, improve osmotic regulation and strengthen antioxidant defenses of tomato seeds, thus contributing to enhanced growth and tolerance to salt stress.

Based on this premise and given both the economic importance of pepper and its sensitivity to salt stress, we propose that a bioproduct derived from tomato cell cultures, enriched with bioactive compounds, represent a promising and sustainable strategy to enhance salinity tolerance during seed germination. We hypothesize that its application as seed priming could improve the germination process, activating antioxidant mechanisms that mitigate the detrimental effects of salt stress. This approach could not only improve germination performance under adverse conditions but also provide a reproducible and environmentally friendly solution for strengthening crop resilience, contributing to the development of next generation biostimulants for sustainable agriculture.

## 2 Materials and methods

**Figure 1:**
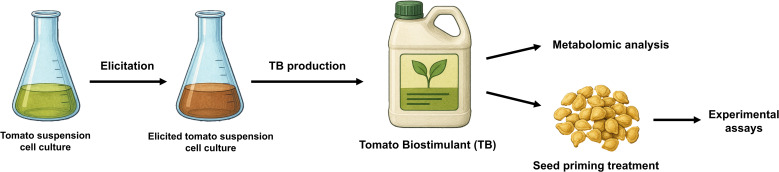
Schematic representation of the experimental design and procedure. TB, tomato biostimulant.

The experimental design has been summarized in a graphical overview in **Figure 1**.

### 2.1 Production of tomato biostimulant

A previously stablished tomato (*Solanum lycopersicum* L. cv. MicroTom) cell line was grown in a culture media optimized for biomass production, and its maintenance was carried out by periodic subcultures every 15 days in liquid Murashige and Skoog basal culture medium (Murashige and Skoog, 1962), supplemented with Morel vitamins (Morel and Wetmore, 1951), 0.8 mg L^-1^ naphthalenacetic acid, 0.1 mg L^-1^ kinetin, 0.25 g L^-1^ casein hydrolysate, and 30 g L^-1^ sucrose at pH 5.8, as described in Briceño et al. (2012) and Sabater-Jara et al. (2022). In order to produce the tomato biostimulant (TB), 15-day-old tomato cell cultures were elicited with 25 mM Me-β-cyclodextrins and 100 µM methyljasmonate for 7 days (Briceño et al., 2012; Sabater-Jara et al., 2022). Non-elicited cell cultures were used as control. Subsequently, elicited cell cultures were homogenized for 5 min with a blender, and a board-spectrum preservative based on a combination of 0.05% (w/v) sodium benzoate and 0.1% (w/v) potassium sorbate was added for the microbiological control of the bioproduct over time. Three different batches of the resulting final bioproduct were analyzed based on the characteristic physical-chemical parameters (pH, conductivity, °Brix and total phenolic content) and used for seed treatment. Total phenolic content was measured by a Folin-Ciocalteu assay as described in (Martí-Guillén et al., 2025b).

### 2.2 Metabolomic characterization of tomato biostimulant

#### 2.2.1 Bioactive compound extraction

Frozen cell lysates (15 mL) were homogenized in 80% (v/v) methanol (1:1, v/v) using a porcelain mortar and pestle under cold conditions. The homogenates were centrifuged at 13,600×*g* for 15 min. The resulting supernatants were subjected to liquid-liquid extraction with ethyl acetate (1:1, v/v) performed twice to obtain bioactive compounds. The combined organic phases were evaporated to dryness under reduced pressure at 40 °C, using a rotary evaporator (Heidolph, Germany). The dried residues were reconstituted in 1 mL of methanol, filtered by 0.22 µm nylon filters and stored at -20 °C until further analysis.

#### 2.2.2 Untargeted metabolomic analysis

Untargeted metabolomic analysis was performed following the protocol described by Zhang et al. (2021). Control and elicited tomato extracts were analyzed in triplicate using an HPLC-MS system composed of an Agilent 1290 Infinity II Series HPLC (Agilent Technologies, Santa Clara, CA, USA) equipped with an Automated Multisampler and a High-Speed Binary Pump, coupled to an Agilent 6550B Q-TOF Mass Spectrometer via an Agilent Jet Stream Dual electrospray (AJS-Dual ESI) interface. Instrument parameters for both HPLC and Q-TOF were configured using MassHunter Workstation Data Acquisition software B.08.00. Samples were separated using an Agilent Zorbax Eclipse Plus column (2.1 × 50 mm, 1.8 µm) at a flow rate of 0.2 mL min^-1^. Analytical blank consisted of LC/MS-grade methanol. Compound separation was achieved using a gradient elution of solvent A (MilliQ water with 0.1% formic acid) and solvent B (acetonitrile with 0.1% formic acid), ranging from 6% to 94% B.

The mass spectrometer operated in Auto MS/MS positive and negative mode. Data were acquired in the 50–1200 m/z range using 2 GHz extended dynamic range in High Resolution mode, with acquisition rates of 1 spectrum s^-1^ for MS and 3 spectra s^-1^ for MS/MS. Ten precursors per cycle were selected based on intensity (minimum peak height of 1000 counts and relative threshold of 0.001%), with active exclusion after two spectra. Collision energies of 10, 20, and 40 eV were applied for fragmentation. Reference masses at 121.0509 and 922.0098 m/z were used for real-time mass correction.

Feature extraction and peak alignment were performed using MS-DIAL (https://systemsomicslab.github.io/compms/index.html). Data interpretations were performed using Metaboanalayst platform (https://www.metaboanalyst.ca/MetaboAnalyst/home.xhtml). Multivariate analysis including unsupervised principal component analysis (PCA) and supervised orthogonal partial least squares discriminant analysis (OPLS-DA) were used to assess group separation, with model quality evaluated via R²Y and Q² metrics (Q² > 0.5 considered acceptable). Discriminant metabolites were identified using the Variable Importance in Projection (VIP) method, applying a threshold of VIP > 1. Statistical significance of selected features was assessed by *t*-test and fold change analysis, expressed as log_2_(FC) comparing elicited versus control groups (Corrado et al., 2021). Compound annotation focused on discriminant features from VIP analysis considering isotopic patterns, including monoisotopic mass, spacing, and relative abundance and MS/MS fragmentation pattern. Only annotations with a confidence score above 90% were retained. According to COSMOS standards in metabolomics, the annotation process adhered to level 2 and 3 of confidence in annotation (putatively and tentative identified metabolites) (Salek et al., 2013). Spectral libraries used included the Fiehn/Vaniya Natural Product Library (https://mona.fiehnlab.ucdavis.edu/), BMDMS-NP, and Bioinformatics & Molecular Design Research Center Mass Spectral Library-Natural Products (BMDMS-NP). Final annotations were manually cross-checked against a curated tomato-specific database to ensure biological relevance. Chemical class enrichment analysis was performed applying a hypergeometric test to evaluate the over-representation of putatively annotated compounds within predefined chemical classes.

### 2.3 Plant material, seed priming and germination tests

For all the experiments, commercial pepper seeds (*Capsicum annum* L. cv. Padrón) purchased from Ramiro Arnedo S.A. (La Rioja, Spain) were used.

#### 2.3.1 Evaluation of seed germination under salt stress conditions

Pepper seeds were germinated in Petri dishes containing sterile filter paper discs moistened with 5 mL of autoclaved distilled water, which served as the control treatment. To induce salt stress, the filter paper was moistened with 5 mL of NaCl solutions at concentrations of 50, 100, 125, 150, or 200 mM. The plates were incubated in darkness at 25 °C for 11 days. Four replicates, each one consisting in 25 seeds per treatment, were used in all experiments.

Germination was monitored daily for 11 days by counting the number of seeds with visible radicle emergence (at least 1 mm), which was used as the criteria for germination. At the end of the experimental period, the fresh weight (FW) of the germinated seedlings was recorded.

The parameters of Final Germination Percentage (FGP), Mean Germination Time (MGT), Germination Index (GI) and Vitality Index (VI) were calculated as described in Martí-Guillén et al. (2025b).

#### 2.3.2 Evaluation of tomato biostimulant effect under salt stress conditions

Prior to seed priming with the biostimulant, the optimal priming duration was determined by monitoring the variation in seed moisture content (MC) over time. Independent batches of 100 mg of seeds were soaked in distilled water for 0.5, 1, 2, 4, 6, 8, 24, and 48 h under orbital shaking at 200 rpm, in darkness, and at room temperature. After each soaking period, seed FW and dry weight (DW) were recorded, and MC was calculated as described by Martí-Guillén et al. (2025b).

For seed priming, pepper seeds were soaked in TB solutions at three concentrations (v/v): 10% (TB10), 1% (TB1), and 0.1% (TB0.1), under the same conditions described above. Hydropriming (HP) treatment, consisting in soaking seeds in distilled water, was used as a hydration control. Following incubation, seeds were air-dried in a ventilated oven at room temperature until their initial MC was restored. Seeds that did not undergo any priming treatment (no priming, NP) were used as an additional control.

To evaluate the effect of TB priming under salt stress, seeds were germinated in Petri dishes containing sterile filter paper discs moistened with 5 mL of either autoclaved distilled water (control) or 125 mM NaCl (salt stress). The dishes were incubated in darkness at 25 °C for 11 days. Each treatment consisted of four replicates of 25 seeds per plate. Germination was monitored daily by counting the number of seeds with visible radicle emergence (at least 1 mm) to calculate the germination parameters: FGP, MGT, GI and VI, as previously described. After 11 days of incubation, seedlings were harvested, and their FW was recorded. The samples were immediately frozen in liquid nitrogen and stored at -80 °C until further analyses, within a week of sampling.

### 2.4 Determination of oxidative stress markers

#### 2.4.1 Determination of H_2_O_2_ content

For the determination of hydrogen peroxide (H_2_O_2_) concentration, the method described by Junglee et al. (2014) with minor modifications was used. Briefly, previously grounded plant material was homogenized in 0.1% (w/v) trichloroacetic acid (TCA) (4:1, w/v) and centrifuged at 14600×*g* for 15 min, recovering the supernatant. The reaction was triggered by adding 50 µL of 50 mM potassium phosphate buffer pH 7.0, 100 µL of 0.1% (w/v) TCA and 250 µL of 1 M KI to 100 µL of extract and incubated at room temperature and in the dark for one hour. The absorbance was measured spectrophotometrically at 390 nm and the H_2_O_2_ concentration was determined using a H_2_O_2_ standard curve ranging from 0 to 500 µM.

#### 2.4.2 Determination of antioxidant enzymes activities

The protein extract was made from previously grounded plant material homogenized (1:2, w/v) in 50 mM potassium phosphate buffer pH 7.0, including 0.1 mM EDTA, 0.1% (v/v) Triton X-100 and 0.05% (w/v) PVP. The mixture was centrifuged for 15 min at 14600×*g* and 4 °C, and the supernatants recovered. Protein concentration was estimated by the Bradford colorimetric method (Bradford, 1976), using different concentrations of BSA (ranging from 0 to 0.6 mg mL^-1^) as standard curve.

POX activity was determined using the protocol described in Ferrer et al. (1990), with minor modifications. 10 µL of the enzyme extract were added to 225 µL of 50 mM sodium acetate pH 5.0, 2.5 µL of 100 mM 4-methoxy-1-naphtol (4-MN) and 12.5 µL of 10 mM H_2_O_2_. Negative controls without H_2_O_2_ were used. Then, the oxidation of 4-MN was quantified spectrophotometrically at 593 nm. POX activity was calculated in the linear phase of oxidized 4-MN generation and was expressed as nmol oxidized 4-MN min^-1^ mg^-1^ protein. The molar extinction coefficient for oxidized 4-MN of 21 mM^-1^ cm^-1^ was used.

The determination of CAT activity was estimated using the protocol described in (Cho and Park, 2000), with some modifications. For this, 10 µL of the protein extract were added to 190 µL of 50 mM potassium phosphate buffer including 15 mM H_2_O_2_. Negative controls without H_2_O_2_ were used. The disappearance of H_2_O_2_ was quantified spectrophotometrically at 240 nm. CAT activity was calculated in the linear phase of H_2_O_2_ consumption and was expressed as mmol H_2_O_2_ min^-1^ mg^-1^ protein. The molar extinction coefficient for H_2_O_2_ of 39.58 M^-1^ cm^-1^ was used.

All spectrophotometric measurements were carried out on a FLUOstar omega microplate reader (BMG LABTECH, Ortenberg, Germany) and three biological replicates were used.

### 2.5 Statistical analysis

In addition to the statistical analysis performed to untargeted metabolomics analysis, seed’s parameters data analysis was developed using SPSS software package (SPSS Inc., Chicago, USA) version 28.0. One-way analysis of variance (ANOVA) was performed, followed by Duncan’s test, and p-values < 0.05 were considered as statistically significant. Principal Component Analysis (PCA) was developed using MiniTab software package (Minitab Inc., Pennsylvania, USA) version 21.0.

## 3 Results

Before performing the evaluation of the biostimulant effect of TB under salt stress conditions, biochemical and metabolomic characterizations of the TB were carried out. Evaluating 3 different batches of TB, the results showed an increase in total phenolic content (TPC) up to 120.57 ± 3.61 μg mL^-1^. In contrast, in the resulting bioproduct in the absence of elicitation, the results showed a TPC of 65.25 ± 7.44 μg mL^-1^. The rest of the physical-chemical parameters analyzed in the TB determined a pH of 5.72, a quantity of soluble sugars of 5.0°Brix, and a conductivity of 3.92 mS cm^-1^ (**Table 1**).

**Table 1:** Biochemical characterization of TB.

	Non-elicited tomato cell cultures	Elicited tomato cell cultures (TB)
pH	6.11	5.72
°Brix	1.5	5.0
Conductivity (mS cm^-1^)	2.87	3.92
TPC (μg GAE mL^−1^)	65.25 ± 7.44	120.57 ± 3.61

TB, tomato biostimulant; TPC, total phenolic content; GAE, gallic acid equivalents.

Given the results regarding the increase in TPC, in order to elucidate the metabolites accumulated due to the elicitation process, an untargeted metabolomic analysis comparing elicited and non-elicited tomato suspension cell cultures was performed.

### 3.1 Untargeted metabolomics analysis of tomato suspension cell cultures

**Figure 2:**
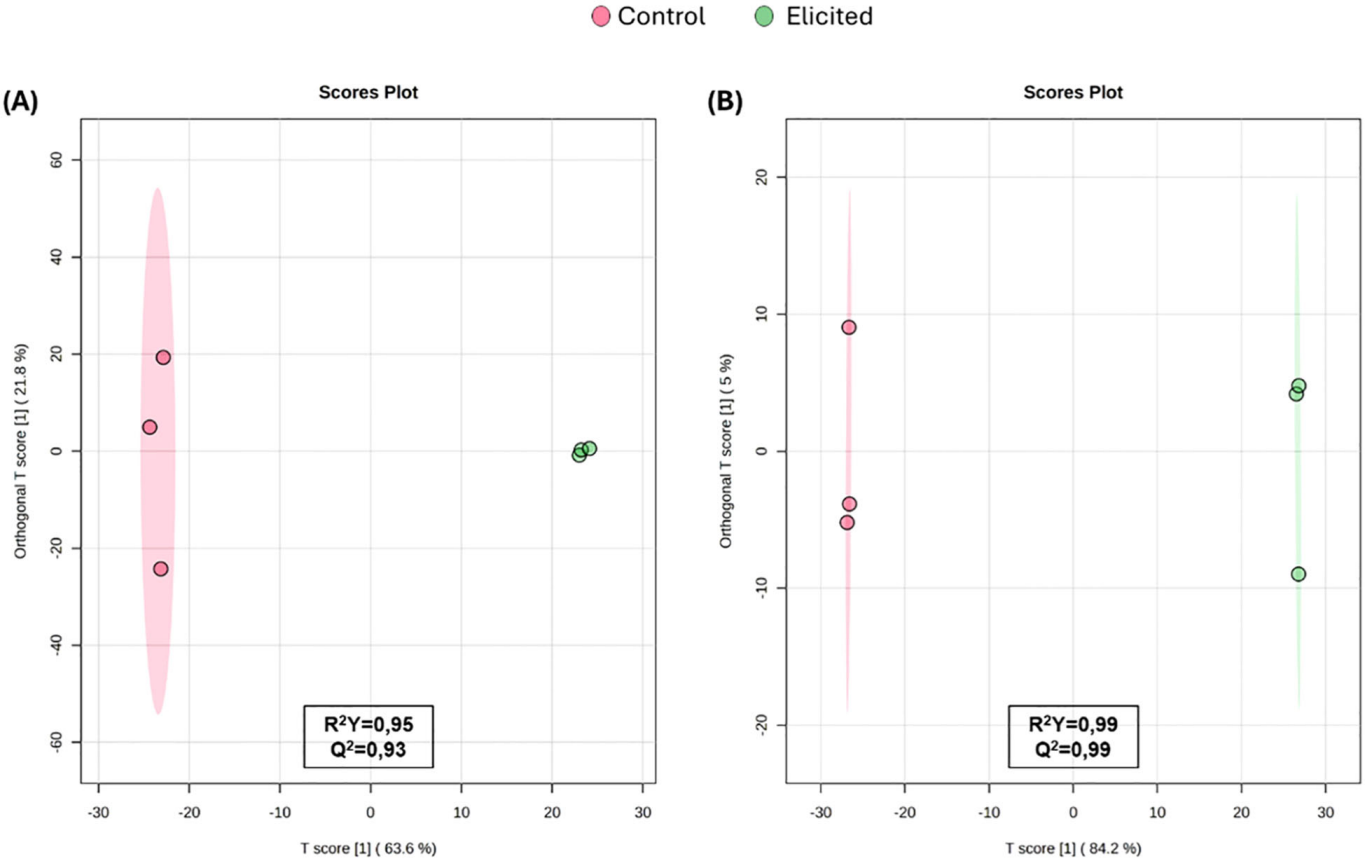
Orthogonal Partial Least Squares Discriminant Analysis (OPLS-DA) built using the metabolic fingerprints of tomato extracts analyzed by Liquid Chromatography–High Resolution Mass Spectrometry (LC-HRMS) in positive **(A)** and negative **(B)** electrospray ionization (ESI) modes.

Untargeted metabolomics analysis in the methanolic extracts identified over 1000 features in both positive and negative electrospray ionization (ESI) modes (**Supplementary Table 1**). Since these ionization modes preferentially detect different metabolites, datasets were processed independently. Metabolic fingerprinting approach was used to investigate if the differences observed in extract effects were caused by changes in chemical composition resulting from elicitation. Then, the multivariate statistics were applied to explore sample clustering and enhance data visualization. The unsupervised PCA and hierarchical cluster analysis (**Supplementary Figure 1**) revealed a clear separation between elicited and non-elicited samples. Subsequently, supervised multivariate analysis using OPLS-DA was applied to confirm the metabolic impact of elicitation and its influence on extract composition. The resulting score plots demonstrated a clear separation of samples according to treatment, indicating that elicitation led to substantial metabolic changes (**Figure 2**). Elicited and non-elicited samples clustered distinctly, confirming the presence of treatment-dependent metabolic profiles. The OPLS-DA models exhibited robust performance, with high goodness-of-fit and predictive ability parameters (R²Y > 0.95 and Q² > 0.99) in both ESI^+^ and ESI^-^ modes. Given the clear discrimination observed, the models were further to identify the most discriminant metabolites contributing to sample separation based on their composition.

To this end, the VIP approach was employed to select metabolites with the highest contribution, using a threshold of VIP > 1. Additionally, FC analysis was performed to identify relative differences in the abundance of each selected marker between experimental groups. These combined strategies allowed the identification of key metabolites associated with the elicitation treatment (**Supplementary Table 2**).

The VIP analysis identified 272 and 264 discriminant metabolites in ESI^−^ and ESI^+^ modes, respectively. Among these, 159 (ESI^−^) and 113 (ESI^+^) features showed increased accumulation in elicited samples compared to controls, while 113 and 151 metabolites, respectively, were found to be decreased (**Supplementary Table 2**). These results indicate that elicitation implied a clear impact on the metabolic profile of tomato extracts, with a substantial number of features differentially accumulated in response to treatment. Moreover, the putative chemical classification of the discriminant features revealed that phenolic compounds, terpenoids, and fatty acids and derivatives were among the most represented classes, suggesting that these metabolic pathways are preferentially modulated by the elicitors applied.

Further, a subset of 21 compounds was putatively annotated (**Supplementary Table 3**). To better interpretated the annotated compounds these 21 metabolites were subjected to chemical class enrichment analysis.

**Figure 3:**
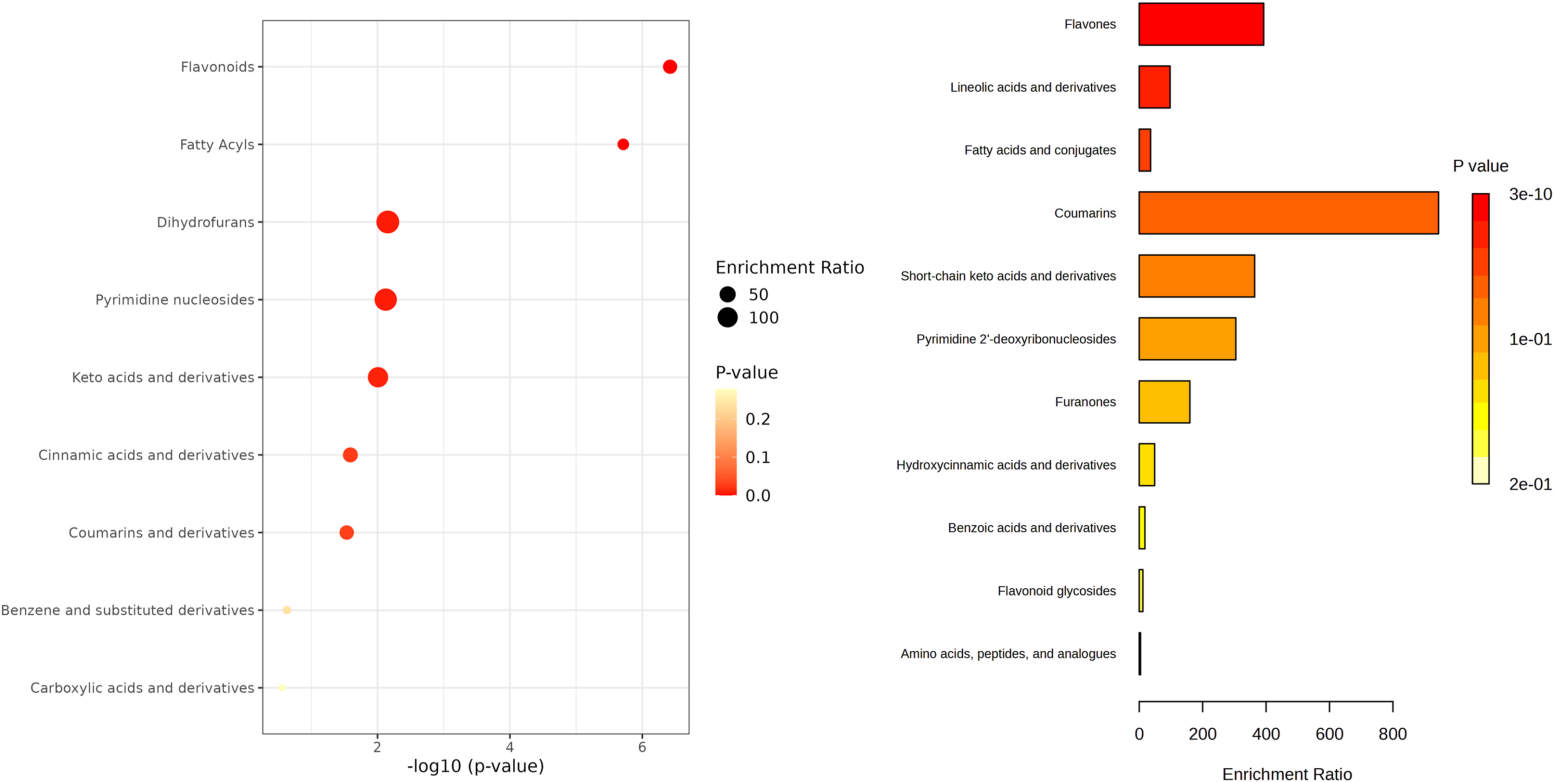
Dot plot of pathway enrichment analysis based on putatively annotated compounds. Circle size represents the enrichment ratio, calculated as the proportion of identified metabolites in each pathway relative to its background frequency. Circle color indicates statistical significance (p-value), with warmer tones reflecting higher significance.

This revealed a significant representation of flavonoids and fatty acyls suggesting that these compound classes are preferentially modulated in tomato extracts subjected to elicitation (**Figure 3**). Within this subset, fatty acids and related derivatives, particularly those associated with linoleic and linolenic acid metabolism, were more abundant in elicited extracts. Regarding phenolics, flavonoids appeared to be more abundant overall, while compounds such as ascorbic acid, coumarin or caffeic acid were predominantly accumulated in the elicited extracts.

Collectively, these results indicated that the elicitation treatment enriched tomato cell suspension in bioactive compounds, which confer this bioproduct a potential biostimulant proprieties. Thus, the effect of TB on ameliorating salt stress in pepper seeds was assessed.

### 3.2 Evaluation of germination parameters of pepper seeds under different salt concentrations

The germination process of pepper seeds was evaluated for 11 days, with the application of different NaCl concentrations, from 0 mM to 200 mM. The results showed that the progressive increase in salt concentration worsened the germination parameters evaluated compared to seeds that germinated without salt stress (0 mM NaCl). Overall, an increase in MGT was observed, as well as a decrease in GI and VI parameters, causing a worsening of the germination process and the development of seedlings with less vitality (**Table 2**).

**Table 2:** Evaluation of germination parameters of pepper seeds with the application of different salt concentrations.

	Germination parameters			
NaCl (mM)	FGP (%)	MGT (day)	GI (seed day^-1^)	VI (mg day^-1^)
0	96 ± 0 a	5,02 ± 0,41 d	4,38 ± 0,09 a	200,38 ± 14,67 a
50	93 ± 4 a	5,75 ± 0,14 c	4,16 ± 0,35 ab	173,22 ± 19,33 b
100	93 ± 6 a	5,71 ± 0,08 c	3,97 ± 0,16 ab	123,10 ± 8,22 c
125	92 ± 1 a	6,25 ± 0,06 b	3,69 ± 0,16 bc	96,49 ± 8,76 d
150	90 ± 4 a	6,62 ± 0,03 b	3,47 ± 0,35 c	72,17 ± 8,74 e
200	48 ± 4 b	8,84 ± 0,31 a	1,55 ± 0,29 d	23,53 ± 4,65 f

Data represents means ± SD (n = 4). Different letters denote significant differences (p < 0.05) in germination parameters between salinity treatments according to Duncan’s test. FGP, Final Germination Percentage. MGT, Mean Germination Time. GI, Germination Index. VI, Vitality Index.

The application of 50 and 100 mM NaCl resulted in a similarly significant increase in MGT values, approximately +14% compared to the absence of salt. However, VI decreased more sharply with the concentration of 100 mM NaCl, decreasing by -39% compared to the absence of salinity, being the reduction with the application of 50 mM NaCl of -14%. Application of higher NaCl concentrations, such as 125 and 150 mM, delayed the MGT value by more than 1 day compared to the absence of salt. Furthermore, they significantly decreased the VI values by -52% and -64%, respectively, as well as the GI values by -16% and -21%, respectively, compared to seeds germinated in the absence of salt. 200 mM NaCl application, although not lethal, increased MGT by almost 4 days and decreased GI and VI by -65% and -88%, respectively, compared to seeds germinated in the absence of salt stress. Furthermore, it was the only salt concentration studied that significantly decreased FGP values, which were reduced by half compared to seeds germinated in the absence of salt stress (**Table 2**).

Therefore, the concentration of 125 mM NaCl was selected since it is a moderately harmful concentration for the germination process of pepper seeds but allows its study because it is not extremely severe for the process.

### 3.3 Evaluation of the biostimulant effect of tomato biostimulant under salt stress conditions

The optimal seed imbibition time was determined prior to seed priming by studying the variation in seed MC over time, similarly to the process described by Martí-Guillén et al. (2025a). As a result, the optimal priming time for pepper seeds was determined to be 2 h, since it allows for an appropriate increase in seed MC, but prevents the irreversible start of the germination phase.

**Figure 4:**
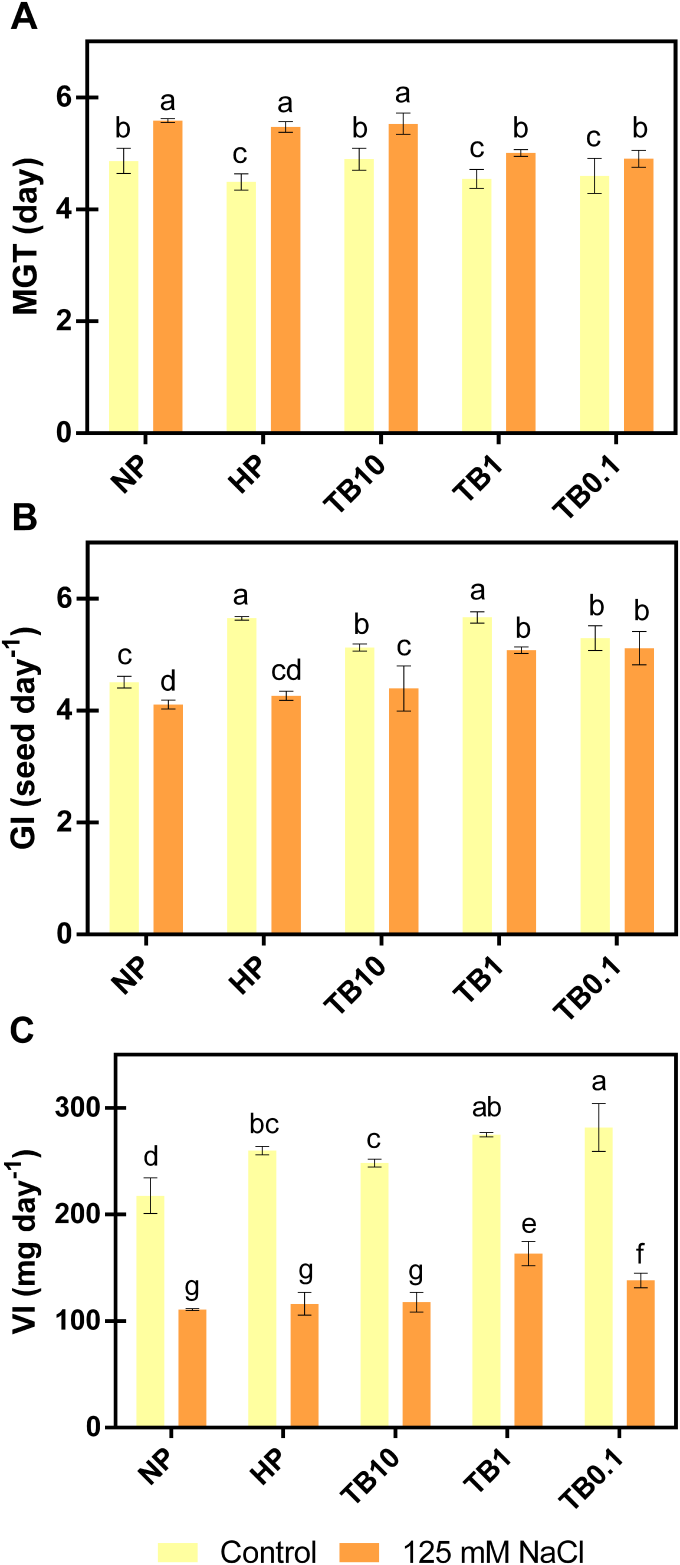
Effect of TB priming treatments in germination parameters of pepper seeds germinated under absence or presence of salinity conditions (125 mM NaCl): MGT **(A)**, GI **(B)** and VI **(C)**. Columns represent mean ± SD (n = 4). Bars with different letters show significant differences (p < 0.05) between the treatments according to Duncan’s test. MGT, Mean Germination Time; GI, Germination Index; VI, Vitality Index; NP, no priming; HP, hydropriming; TB, tomato biostimulant at 10, 1 and 0.1% (v/v).

Once the optimal priming time was determined, different batches of pepper seeds were primed with 3 different doses of TB: TB10, TB1 and TB0.1. In order to evaluate the biostimulant effect of TB in pepper seeds under salt stress conditions, seeds were germinated in the absence or presence of 125 mM NaCl. The results showed that priming pepper seeds with TB at any dose improved germination parameters evaluated in the absence of salt. However, most of these improvements were not significantly different from the HP treatment. When germination parameters were evaluated under salt stress conditions, 125 mM NaCl increased MGT value, as well as decreased the GI and VI values with respect to seeds germinated in the absence of salt. In this context, HP treatment did not differ significantly from the no-priming treatment (NP). However, TB1 and TB0.1 doses significantly improved all germination parameters evaluated with respect to NP and HP (**Figure 4**).

Regarding MGT (**Figure 4A**), under salt stress, TB1 dose reduced its value by -10% and -8% with respect to NP and HP, respectively. Comparably, the TB0.1 dose achieved similar results, decreasing MGT value by -12% and -10% with respect to NP and HP. Regarding the GI (**Figure 4B**), with the application of 125 mM NaCl, both TB1 and TB0.1 doses increased the value by approximately +25% with respect to NP and +20% with respect to HP. Regarding the VI (**Figure 4C**), this was the parameter most affected by the saline treatment, especially due to the decrease in the FW of the developed seedlings. However, treatment with TB doses ≤ 1% (v/v) partially reversed the drastic consequences of salt stress. TB0.1 managed to increase its value by +26% and +19% compared to NP and HP, respectively. Similarly, TB1 reached the highest values for this parameter, increasing its value by +47% and +41% compared to NP and HP, respectively.

Based on the results obtained, TB1 and TB0.1 doses significantly improved germination parameters compared to the NP and HP treatments, especially TB1 dose, which achieved the greatest partial reversal of VI, the most sensitive parameter to salt stress. Therefore, the TB1 dose was selected for further analysis.

### 3.4 Evaluation of the effect of tomato biostimulant and salt stress on oxidative stress markers in pepper seedlings

To evaluate the biostimulant effect of TB on salt stress tolerance in pepper seedlings, various oxidative stress markers related to H_2_O_2_ content and the activity of key detoxification enzymes were determined.

**Figure 5:**
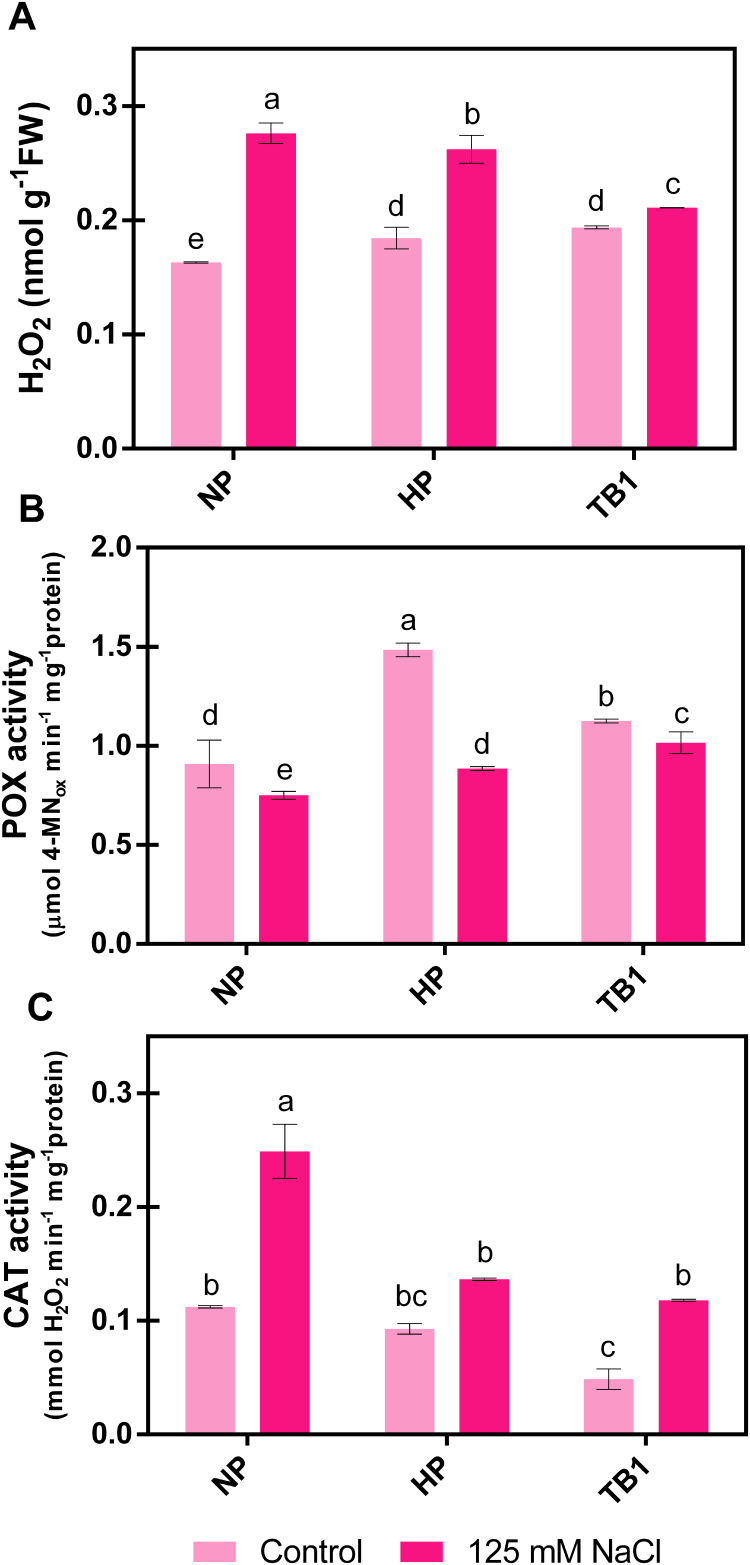
Effect of TB priming treatment on H_2_O_2_ content **(A)**, and POX **(B)** and CAT **(C)** activities of pepper seedlings germinated under absence or presence of salinity conditions (125 mM NaCl). Columns represent mean ± SD (n = 3). Bars with different letters show significant differences (p < 0.05) between the treatments according to Duncan’s test. NP, no priming; HP, hydropriming; TB1, tomato biostimulant at 1% (v/v).

H_2_O_2_ is a signaling molecule under normal physiological conditions. However, under stress conditions, its content increases, potentially damaging cellular components and biomolecules. The results showed that the exposure to 125 mM NaCl increased H_2_O_2_ content in all treatments, but particularly in the untreated seeds (NP), where their concentration increased by +69% compared to NP seeds in absence of salinity conditions (**Figure 5A**). This increase was significantly reduced with both HP and TB1 treatments. However, the TB1 treatment achieved the greatest reduction in H_2_O_2_ content under salt stress, reducing it by -24% and -19% compared to NP and HP, respectively (**Figure 5A**).

The capacity to detoxify H_2_O_2_ was assessed by determining the activities of its main scavenger enzymes, POX and CAT. As shown in **Figure 5B**, with the application of 125 mM NaCl, POX activity was significantly reduced in untreated seeds (NP) compared to the absence of salinity (0 mM NaCl). However, with both priming treatments, its activity was not compromised by salinity, increasing by +18% in the case of HP and +35% in the case of TB1, compared to NP. Regarding CAT activity (**Figure 5C**), salt stress increased its value more than double in untreated seeds (NP) compared to the unstressed seeds. In contrast, with the priming treatments, the results showed a lower promotion of CAT activity under salt stress conditions, reducing this value by approximately -50% compared to NP seeds, achieving an activity value similar to that present in NP seeds germinated under non-salinity conditions. No significant differences were observed between the CAT activity values of HP and TB1 in the presence of 125 mM NaCl.

**Figure 6:**
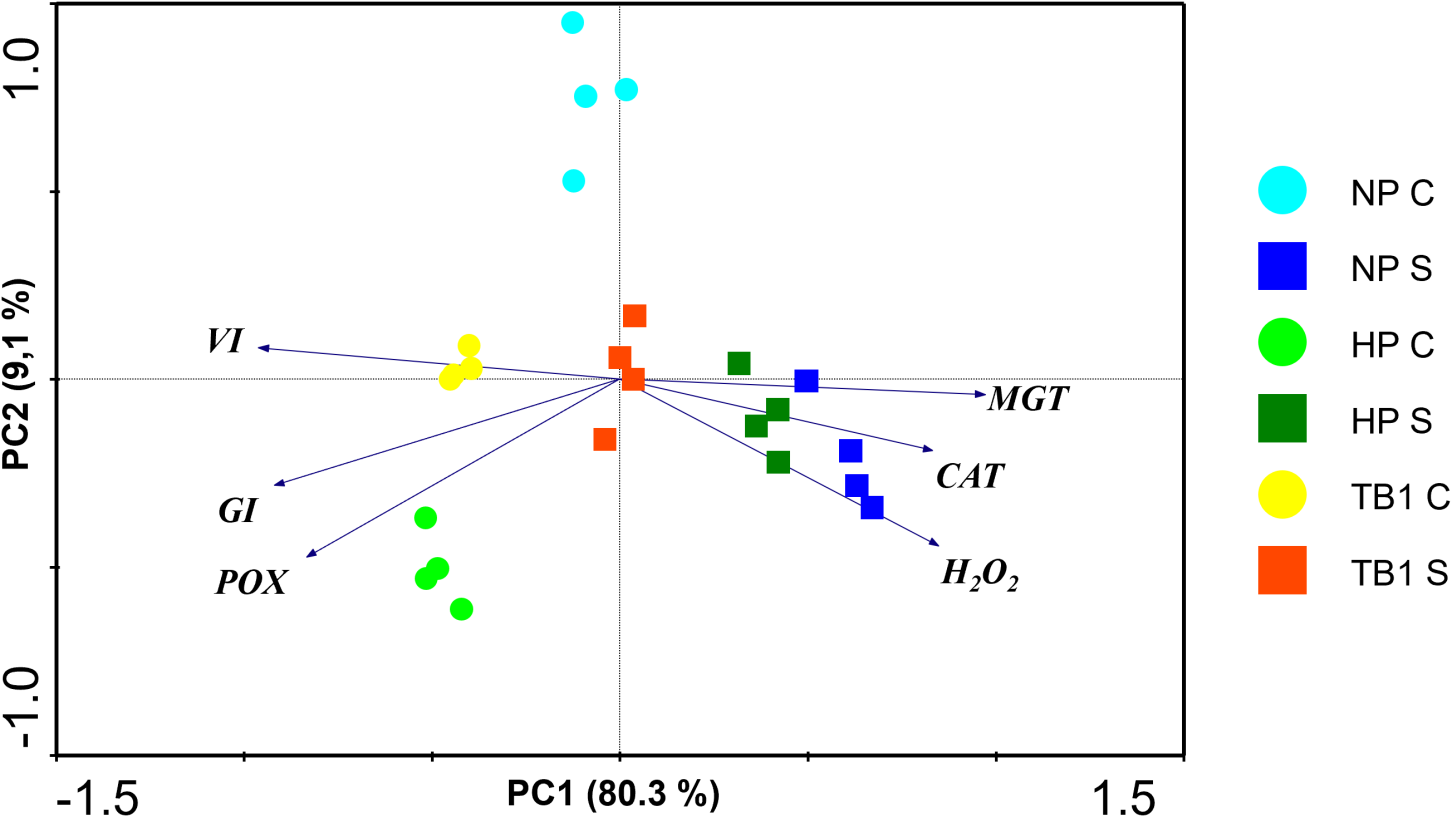
Principal component analysis (PCA) based on the correlation matrix applied to germination parameters and oxidative stress markers data sets. Seeds germinated in absence of salinity conditions (C) and in presence of salinity conditions (S, 125 mM NaCl) are represented by circles and squares, respectively. Each variable is represented by an arrow. The longer the length of the arrow, the greater its contribution to a given component. Angles between arrows indicate the degree of correlation among variables. The smaller the angle, the higher the correlation. PC, principal components 1 and 2; NP, no priming; HP, hydropriming; TB, tomato biostimulant at 1% (v/v).

A principal component analysis (PCA) of all conditions was carried out in order to explore the physiological and biochemical profiles in pepper seedlings after treatment with TB and exposed to salinity conditions, integrating all data obtained. As can be seen in **Figure 6**, the first principal component (PC1), which explained 80.3% of the total variance, was mainly influenced by parameters related to germination parameters, MGT on the positive side of the X-axis, and GI and VI on the negative side. The second principal component (PC2), which explained 9.1% of the total variance, was influenced mainly by oxidative stress markers on the negative side of the Y-axis. Furthermore, the projection of the samples on the axes showed a clear separation based on the application of salt stress. The graph clearly distinguishes the groups subjected to salt stress, located on the right side, separating the untreated seeds (NP S) from those that received hydropriming (HP S), and even more from those treated with TB1 (TB1 S). On the other hand, the seeds that were not subjected to salt stress were located on the left side of the graph, showing a clear distinction between the three population groups. Among the groups germinated under salt stress, the seeds treated with TB1 (TB1 S) was the closest to the groups of seeds that were not exposed to salinity conditions, demonstrating that the treatment with TB1 partially reversed the alterations induced by salt stress in pepper seedlings.

## 4 Discussion

The effects derived from climate change arise, such as the salinization of arable lands, are reducing the yield of crops that sustain the food supply of the world’s population (Mittler et al., 2025; Zhang et al., 2025). As an alternative, techniques to improve crop yield under these adverse conditions, especially those that do no significant harm (DNSH) to the environment, are emerging to avoid compromised food security. Seed priming, especially with hPDBs, stands out as an alternative technology to promote germination and early seedling development, especially under edaphoclimatic pressure (Martínez-Lorente et al., 2024).

In this context, our research has focused on producing a novel type of biostimulant never before described and derived from elicited tomato cell cultures, with the subsequent evaluation of its effect on pepper seeds subjected to salt stress during the germination process.

Tomato fruit has antioxidant properties due to its rich composition in bioactive compounds. Among the bioactive compounds that characterize this fruit, its richness in carotenoids, vitamins (mainly ascorbic acid), and phenolic compounds (both phenolic acids and flavonoids) stands out (Slimestad and Verheul, 2009; Chaudhary et al., 2018). In our study, we have used a cell line generated from immature tomato fruits that, under elicitation treatments, is capable of producing a broad spectrum of specialized metabolites in a controlled and scalable manner, as described by Briceño et al. (2012). In fact, according to the results of the untargeted metabolomic analysis, the elicitation process enriched tomato cell cultures in fatty acids and phenolic compounds, increasing the content in flavonoids and other bioactive metabolites such as caffeic acid, coumarin and ascorbic acid, among others (**Figure 3**). These metabolites have been described with biostimulant potential, as they are potent antioxidants which can also modulate plant defense responses. In fact, their role as biostimulants increasing abiotic stress tolerance has been described in multiple studies (Alves et al., 2021; Parvin et al., 2021; Dmytryk et al., 2022; Mughal et al., 2024; Ramzan et al., 2024; Rao and Zheng, 2025). Therefore, given the enrichment of elicited tomato cell suspensions in these compounds, we used the resulting bioproduct, TB, as a seed priming agent for pepper seeds and evaluated its effectiveness ameliorating salt stress.

Salinity conditions compromise the germination process, especially due to the ROS overproduction they induce, such as H_2_O_2_. This generates oxidative damage to cellular components and biomolecules, which compromises the structural integrity and physiological development of plants. However, enzymatic and non-enzymatic antioxidant mechanisms, such as the activity of detoxifying enzymes and the accumulation of antioxidant molecules, can scavenge ROS overproduction and alleviate oxidative damage (Martí-Guillén et al., 2022; Martínez-Lorente et al., 2022).

In this context, the evaluation of the germination process determined that HP treatment managed to improve germination parameters, compared to the absence of priming treatment, in the absence of salinity. However, with 125 mM NaCl application, HP failed to significantly improve almost any of the parameters evaluated, compared to non-primed seeds. In contrast, TB, especially at the 1% (v/v) dose, retained the same advantages as the HP treatment in the absence of salinity, and also managed to improve most of the parameters evaluated in the presence of salinity (**Figure 4**). Regarding oxidative stress markers under salinity conditions, HP treatment significantly decreased H_2_O_2_ content, sustained POX activity, and did not exponentially promote catalase activity. However, these improvements induced by HP compared to NP under salinity conditions were significantly lower than those of TB treatment, which achieved the greatest quenching of salt stress-induced H_2_O_2_ (-24%), especially by promoting POX activity up to +35% (**Figure 5**).

Numerous studies have demonstrated the ability of phenolic compounds-based biostimulants to alleviate the negative effects of salt stress by reducing ROS levels. These compounds can directly scavenge ROS and stimulate plant adaptation mechanisms (Rao and Zheng, 2025). Among the phenolic compounds enriched in TB, coumarin, caffeic acid and flavonoids stand out. Coumarin has been gaining attention as a compound capable of interacting with different enzymes and receptors, modulating their activity (Annunziata et al., 2020). Concretely, Parvin et al. (2021) demonstrated that coumarin treatment increased salinity tolerance in tomato plants, another crop from the Solanaceae family, by reducing H_2_O_2_ levels and oxidative damage through the promotion of POX activity. Caffeic acid has also been linked to a better abiotic stress tolerance due to its antioxidant role and its capacity to upregulate the activity of antioxidant enzymes (Mughal et al., 2024). Biostimulants enriched in caffeic acid have been shown to increase salt stress tolerance when applied as seed priming (Bajwa et al., 2018). In fact, the combined treatment of caffeic acid and kaempferol, a flavonoid which is also enriched in our TB, managed to mitigate salt stress in other Solanaceae crops, such as potato, decreasing H_2_O_2_ levels and modulating antioxidant enzyme activity (Ramzan et al., 2024). Our bioproduct also stands out for its high content in ascorbic acid, which is mainly known as an antioxidant, although it can stimulate other physiological processes. In recent years, numerous studies have demonstrated the positive effect of ascorbic acid seed priming for improving the germination process (Terzaghi and De Tullio, 2023). Recent studies by Alves et al. (2021) showed that tomato seeds primed with ascorbic acid exhibited an enhanced salinity tolerance due to an increase in antioxidant defense systems, including a higher POX activity, which lead to a lower H_2_O_2_ content. The untargeted metabolomic analysis also described an accumulation of fatty acids in TB, which have recently also been associated with biostimulant properties when applied as seed treatment, improving seed germination and seedling growth (Dmytryk et al., 2022; Canelas et al., 2024).

These results demonstrate that HP treatment can accelerate the germination process by activating pre-germinative metabolism. However, under salt stress conditions, these effects fail to translate into improvements in the germination process compared to the absence of priming treatment. However, seed priming treatment with TB at a dose of 1% (v/v) significantly improves salt stress tolerance, resulting in the development of seedlings with improved physiological quality and less oxidative damage. Therefore, TB is established as a novel type of hPDB effective in providing salt stress tolerance to pepper seeds due to its composition rich in bioactive compounds, which is capable of inducing the activation of antioxidant defense mechanisms. However, given the genetic and morphological heterogeneity of seeds from other plant species compared to pepper seeds, these results could differ when applying TB to other varieties, requiring adjustments to its application time or dose to achieve the biostimulant effect. Furthermore, pepper seeds are not only commercialized as seeds but also as seedlings, which are exposed to high soil and climate pressures when transplanted to the field. Therefore, it is necessary to determine whether TB is also effective at different phenological stages of the crop.

Thus, together with other hPDBs developed in our research group, specifically based on elicited cell cultures of grapevine (Martí-Guillén et al., 2025b) and yellow carrot (Martí-Guillén et al., 2025a), TB is incorporated into a novel group of next generation biostimulants based on *in vitro* plant cell cultures with the capacity to induce salinity tolerance in crops of high agronomic interest with minimum ecological impact. In addition, the low doses needed for their efficiency and the minimum processing for their production allow them to be cost-effective for their application in agriculture.

## 5 Conclusions and future perspectives

In this research, we have developed a novel type of biostimulant based on tomato cell cultures elicited with 25 mM Me-β-cyclodextrins and 100 µM methyljasmonate, resulting in a bioproduct enriched in bioactive compounds, especially various flavonoids and fatty acids, as well as coumarin, ascorbic acid and caffeic acid. The use *in vitro* plant cell cultures for producing plant biostimulants has not been described before in literature, and our research group is pioneer in exploring its potential. The application of our bioproduct, especially at a dose of 1% (v/v), demonstrated its biostimulant effect on pepper seeds subjected to salt stress during the germination process, achieving the development of seedlings with greater vigor and better physiological quality, reversing the alterations caused by salt stress. Therefore, our tomato biostimulant (TB) is established as a new type of plant biostimulant with the ability to improve the germination process and early development of pepper seeds under salt stress conditions when applied as seed priming.

New fields of research are opened to understand the underlying mechanisms of action of the biostimulant in improving salt stress tolerance, especially its modulation of plant physiological processes. Furthermore, with the aim of obtaining a greater agronomic impact, it would be necessary to evaluate its effectiveness on other crops of great agronomic interest. These topics will be addressed in future research.

## Data Availability

The original contributions presented in the study are included in the article/**Supplementary Material**. Further inquiries can be directed to the corresponding author/s.

## References

[B1] Acosta J. A. Faz A. Jansen B. Kalbitz K. Martínez-Martínez S. (2011). Assessment of salinity status in intensively cultivated soils under semiarid climate, Murcia, SE Spain. J. Arid Environments 75, 1056–1066. doi: 10.1016/j.jaridenv.2011.05.006

[B2] Albaladejo-Marico L. Gomez-Molina M. Garcia-Ibañez P. Carvajal M. Yepes-Molina L. (2025). Valorization of broccoli by-products: seasonal variations in bioactive compounds and their biostimulant effects on pak choi germination. PloS One 20, e0323848. doi: 10.1371/journal.pone.0323848, PMID: 40373095 PMC12101848

[B3] Alves R. de C. Rossatto D. R. da Silva J.d. S. Checchio M. V. de Oliveira K. R. . (2021). Seed priming with ascorbic acid enhances salt tolerance in micro-tom tomato plants by modifying the antioxidant defense system components. Biocatalysis Agric. Biotechnol. 31, 101927. doi: 10.1016/j.bcab.2021.101927

[B4] Annunziata F. Pinna C. Dallavalle S. Tamborini L. Pinto A. (2020). An overview of coumarin as a versatile and readily accessible scaffold with broad-ranging biological activities. Int. J. Mol. Sci. 21, 4618. doi: 10.3390/ijms21134618, PMID: 32610556 PMC7370201

[B5] Bajwa A. A. Farooq M. Nawaz A. (2018). Seed priming with sorghum extracts and benzyl aminopurine improves the tolerance against salt stress in wheat (*Triticum aestivum* L.). Physiol. Mol. Biol. Plants 24, 239–249. doi: 10.1007/s12298-018-0512-9, PMID: 29515318 PMC5834994

[B6] Belchí-Navarro S. Almagro L. Sabater-Jara A. B. Fernández-Pérez F. Bru R. Pedreño M. A. (2013). Induction of *trans*-resveratrol and extracellular pathogenesis-related proteins in elicited suspension cultured cells of *Vitis vinifera* cv Monastrell. J. Plant Physiol. 170, 258–264. doi: 10.1016/j.jplph.2012.10.003, PMID: 23127362

[B7] Bradford M. M. (1976). A rapid and sensitive method for the quantitation of microgram quantities of protein utilizing the principle of protein-dye binding. Analytical Biochem. 72, 248–254. doi: 10.1016/0003-2697(76)90527-3, PMID: 942051

[B8] Briceño Z. Almagro L. Sabater-Jara A. B. Calderón A. A. Pedreño M. A. Ferrer M. A. (2012). Enhancement of phytosterols, taraxasterol and induction of extracellular pathogenesis-related proteins in cell cultures of *Solanum lycopersicum* cv Micro-Tom elicited with cyclodextrins and methyl jasmonate. J. Plant Physiol. 169, 1050–1058. doi: 10.1016/j.jplph.2012.03.008, PMID: 22608078

[B9] Canelas C. A. Dutra J. Gomes T. Freitas L. Silva D. Azevedo V. . (2024). Use of fatty acids in fertilizer formulation: A systematic review. MPS 4, 1–14. doi: 10.47485/2832-9384.1065

[B10] Chaudhary P. Sharma A. Singh B. Nagpal A. K. (2018). Bioactivities of phytochemicals present in tomato. J. Food Sci. Technol. 55, 2833–2849. doi: 10.1007/s13197-018-3221-z, PMID: 30065393 PMC6045986

[B11] Chen K. Arora R. (2013). Priming memory invokes seed stress-tolerance. Environ. Exp. Bot. 94, 33–45. doi: 10.1016/j.envexpbot.2012.03.005

[B12] Cho U.-H. Park J.-O. (2000). Mercury-induced oxidative stress in tomato seedlings. Plant Sci. 156, 1–9. doi: 10.1016/S0168-9452(00)00227-2, PMID: 10908800

[B13] Corrado G. Lucini L. Miras-Moreno B. Zhang L. El-Nakhel C. Colla G. . (2021). Intraspecific variability largely affects the leaf metabolomics response to isosmotic macrocation variations in two divergent lettuce (*Lactuca sativa* L.) varieties. Plants 10, 91. doi: 10.3390/plants10010091, PMID: 33466229 PMC7824788

[B14] Dmytryk A. Samoraj M. Moustakas K. Witek-Krowiak A. Chojnacka K. (2022). Bioactive fatty acids and compounds from *Spirulina (Arthrospira) platensis*: Potential as biostimulants for plant growth. Sustain. Chem. Pharm. 30, 100899. doi: 10.1016/j.scp.2022.100899

[B15] European Commission (2019). “ The european green deal,” in Communication from the commission to the european parliament, the european council, the council, the european economic and social committee and the committee of the regions ( European Commission, Brussels, Belgium). Available online at: https://eur-lex.europa.eu/legal-content/EN/TXT/?uri=CELEX:52019DC0640.

[B16] FAO (2023). Statistical databases ( Food and Agriculture Organization of the United Nations). Available online at: https://www.fao.org/faostat/en/data (Accessed October 10, 2025).

[B17] Ferrer M. A. Calderón A. A. Muñoz R. Ros Barceló A. (1990). 4-Methoxy-α-naphthol as a specific substrate for kinetic, zymographic and cytochemical studies on plant peroxidase activities. Phytochemical Anal. 1, 63–69. doi: 10.1002/pca.2800010203

[B18] Ibrahim E. A. (2016). Seed priming to alleviate salinity stress in germinating seeds. J. Plant Physiol. 192, 38–46. doi: 10.1016/j.jplph.2015.12.011, PMID: 26812088

[B19] Junglee S. Urban L. Sallanon H. Lopez-Lauri F. (2014). Optimized assay for hydrogen peroxide determination in plant tissue using potassium iodide. Am. J. Analytical Chem. 5, 730–736. doi: 10.4236/ajac.2014.511081

[B20] Majeed A. Muhammad Z. (2019). “ Salinity: A major agricultural problem—Causes, impacts on crop productivity and management strategies,” in Plant abiotic stress tolerance: agronomic, molecular and biotechnological approaches. Eds. Hasanuzzaman M. Hakeem K. R. Nahar K. Alharby H. F. ( Springer International Publishing, Cham), 83–99. doi: 10.1007/978-3-030-06118-0_3

[B21] Martí-Guillén J. M. Martínez-Lorente S. E. Pedreño M.Á. Almagro L. Sabater-Jara A. B. (2025a). “ Biotechnological production of plant bioactive compounds as a strategy to obtain natural plant biostimulants,” in Progress in botany ( Springer, Berlin, Heidelberg), 1–21. doi: 10.1007/124_2025_90

[B22] Martí-Guillén J. M. Martínez-Lorente S. E. Pedreño M.Á. Almagro L. Sabater-Jara A. B. (2025b). Grapevine cell culture-based biostimulant alleviates salt stress in tomato seeds. J. Agric. Food Res. 22, 102145. doi: 10.1016/j.jafr.2025.102145 42537284

[B23] Martí-Guillén J. M. Pardo-Hernández M. Martínez-Lorente S. E. Almagro L. Rivero R. M. (2022). Redox post-translational modifications and their interplay in plant abiotic stress tolerance. Front. Plant Sci. 13. doi: 10.3389/fpls.2022.1027730, PMID: 36388514 PMC9644032

[B24] Martínez-Lorente S. E. Martí-Guillén J. M. Pedreño M.Á. Almagro L. Sabater-Jara A. B. (2024). Higher plant-derived biostimulants: mechanisms of action and their role in mitigating plant abiotic stress. Antioxidants 13, 318. doi: 10.3390/antiox13030318, PMID: 38539851 PMC10967762

[B25] Martínez-Lorente S. E. Pardo-Hernández M. Martí-Guillén J. M. López-Delacalle M. Rivero R. M. (2022). Interaction between melatonin and NO: action mechanisms, main targets, and putative roles of the emerging molecule NOmela. Int. J. Mol. Sci. 23, 6646. doi: 10.3390/ijms23126646, PMID: 35743084 PMC9223470

[B26] Ministerio de Agricultura, Pesca y Alimentación de España (2023). Anuario de Estadística. Available online at: https://www.mapa.gob.es/es/estadistica/temas/publicaciones/anuario-de-estadistica/2023 (Accessed October 10, 2025).

[B27] Mittler R. Karlova R. Bassham D. C. Lawson T. (2025). Crops under stress: can we mitigate the impacts of climate change on agriculture and launch the ‘Resilience Revolution’? Philos. Trans. R. Soc. B: Biol. Sci. 380, 20240228. doi: 10.1098/rstb.2024.0228, PMID: 40439296 PMC12121375

[B28] Morel G. Wetmore R. H. (1951). Fern callus tissue culture. Am. J. Bot. 38, 141–143. doi: 10.1002/j.1537-2197.1951.tb14804.x

[B29] Mughal A. Jabeen N. Ashraf K. Sultan K. Farhan M. Hussain M. I. . (2024). Exploring the role of caffeic acid in mitigating abiotic stresses in plants: A review. Plant Stress 12, 100487. doi: 10.1016/j.stress.2024.100487

[B30] Murashige T. Skoog F. (1962). A revised medium for rapid growth and bio assays with tobacco tissue cultures. Physiologia Plantarum 15, 473–497. doi: 10.1111/j.1399-3054.1962.tb08052.x

[B31] Parvin K. Hasanuzzaman M. Mohsin S. M. Nahar K. Fujita M. (2021). Coumarin improves tomato plant tolerance to salinity by enhancing antioxidant defence, glyoxalase system and ion homeostasis. Plant Biol. 23, 181–192. doi: 10.1111/plb.13208, PMID: 33135242

[B32] Ramzan M. Haider S. T. A. Hussain M. B. Ehsan A. Datta R. Alahmadi T. A. . (2024). Potential of kaempferol and caffeic acid to mitigate salinity stress and improving potato growth. Sci. Rep. 14, 21657. doi: 10.1038/s41598-024-72420-0, PMID: 39294197 PMC11410995

[B33] Rao M. J. Zheng B. (2025). The role of polyphenols in abiotic stress tolerance and their antioxidant properties to scavenge reactive oxygen species and free radicals. Antioxidants 14, 74. doi: 10.3390/antiox14010074, PMID: 39857408 PMC11761259

[B34] Sabater-Jara A. B. Almagro L. Belchí-Navarro S. Ferrer M.Á. Barceló A. R. Pedreño M.Á. (2010). Induction of sesquiterpenes, phytoesterols and extracellular pathogenesis-related proteins in elicited cell cultures of *Capsicum annuum*. J. Plant Physiol. 167, 1273–1281. doi: 10.1016/j.jplph.2010.04.015, PMID: 20594613

[B35] Sabater-Jara A. B. Almagro L. Pedreño M. A. (2014a). Induction of extracellular defense-related proteins in suspension cultured-cells of *Daucus carota* elicited with cyclodextrins and methyl jasmonate. Plant Physiol. Biochem. 77, 133–139. doi: 10.1016/j.plaphy.2014.02.006, PMID: 24589476

[B36] Sabater-Jara A. B. Marín-Marín M. J. Almagro L. Pedreño M. A. (2022). Cyclodextrins increase triterpene production in *solanum lycopersicum* cell cultures by activating biosynthetic genes. Plants 11, 2782. doi: 10.3390/plants11202782, PMID: 36297806 PMC9609435

[B37] Sabater-Jara A.-B. Onrubia M. Moyano E. Bonfill M. Palazón J. Pedreño M. A. . (2014b). Synergistic effect of cyclodextrins and methyl jasmonate on taxane production in *Taxus x media* cell cultures. Plant Biotechnol. J. 12, 1075–1084. doi: 10.1111/pbi.12214, PMID: 24909837

[B38] Salek R. M. Steinbeck C. Viant M. R. Goodacre R. Dunn W. B. (2013). The role of reporting standards for metabolite annotation and identification in metabolomic studies. Gigascience 2, 13. doi: 10.1186/2047-217X-2-13, PMID: 24131531 PMC3853013

[B39] Slimestad R. Verheul M. (2009). Review of flavonoids and other phenolics from fruits of different tomato (*Lycopersicon esculentum* Mill.) cultivars. J. Sci. Food Agric. 89, 1255–1270. doi: 10.1002/jsfa.3605

[B40] Terzaghi M. De Tullio M. C. (2023). Ascorbic acid in seeds, priming and beyond. Seeds 2, 421–435. doi: 10.3390/seeds2040032

[B41] Zhang H. Lang Z. Zhu J.-K. Wang P. (2025). Tackling abiotic stress in plants: recent insights and trends. Stress Biol. 5, 8. doi: 10.1007/s44154-025-00216-x

[B42] Zhang L. Saber F. R. Rocchetti G. Zengin G. Hashem M. M. Lucini L. (2021). UHPLC-QTOF-MS based metabolomics and biological activities of different parts of *Eriobotrya japonica*. Food Res. Int. 143, 110242. doi: 10.1016/j.foodres.2021.110242, PMID: 33992354

