# Supplementary material for: Tomato cell cultures as innovative plant biostimulants: evaluation of its effect on pepper seeds germinated under salinity conditions
Source: Front Plant Sci. 2025 Dec 2;16:1726488. doi: 10.3389/fpls.2025.1726488 (PMC12705415; doi:10.3389/fpls.2025.1726488)
Supplement: Supplementary file 1 [file DataSheet1.docx]

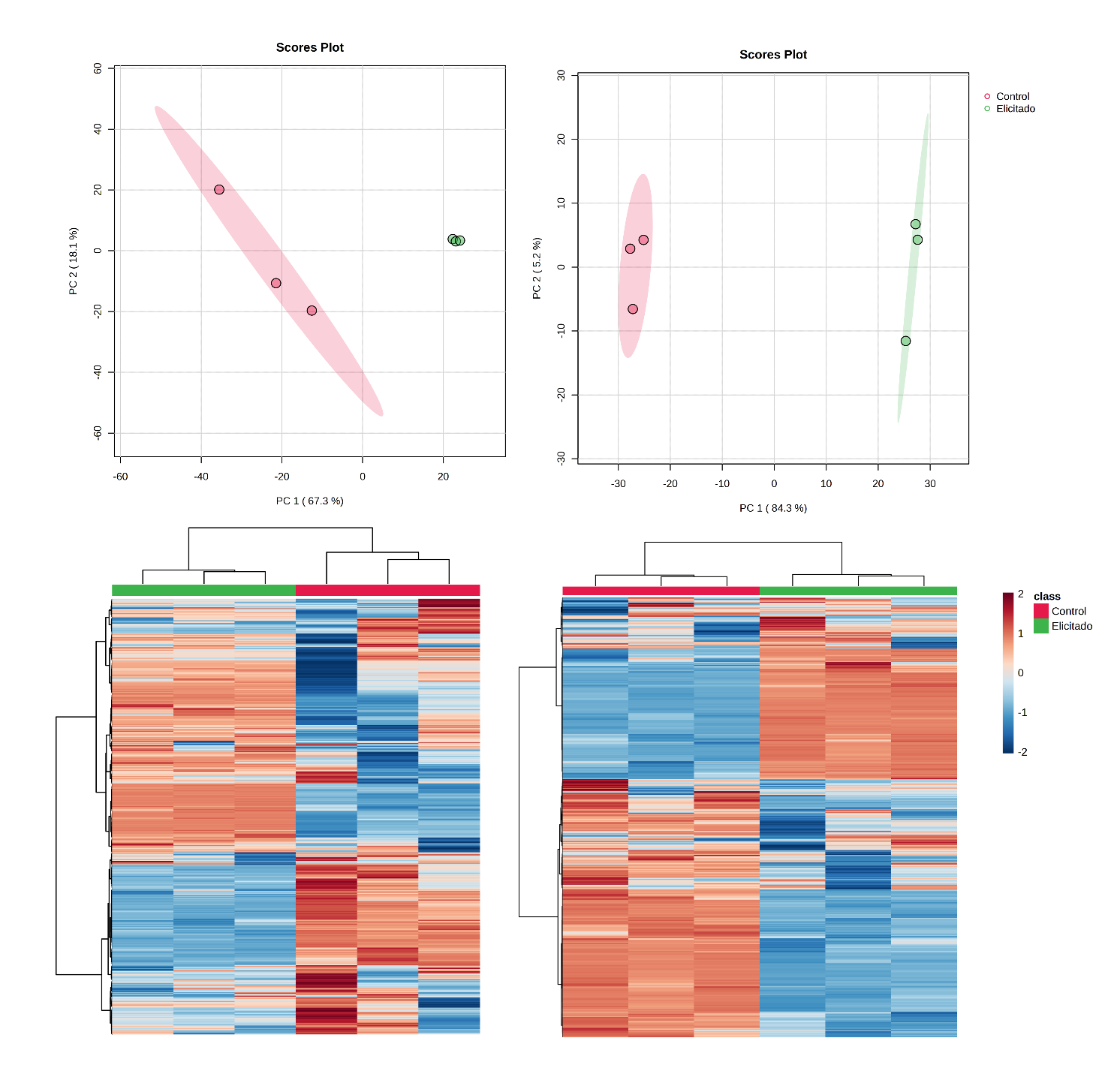
Supplementary Material

**Supplementary Figure 1.** Unsupervised PCA and hierarchical cluster analysis of non-elicited and elicited tomato suspension cell cultures.

**Supplementary Table 1**. Identification of untargeted metabolomics analysis in the methanolic extracts in both positive and negative electrospray ionization (ESI) modes.

**Supplementary Table 2**. Identification of key metabolites associated with the elicitation treatment.

**Supplementary Table 3**. Putative chemical classification of the discriminant features.
